# Prevalence and predictors of bowel dysfunction in a large multiple sclerosis outpatient population: an Italian multicenter study

**DOI:** 10.1007/s00415-021-10737-w

**Published:** 2021-08-04

**Authors:** Bisecco Alvino, Fornasiero Arianna, Bianco Assunta, Cortese Antonio, d’Amico Emanuele, Mataluni Giorgia, Sinisi Leonardo, Spitaleri Daniele, Docimo Renato, Maria Chiara Buscarinu, Mirabella Massimiliano, Sebastiano Giuseppe Crisafulli, Zanghì Aurora, Carolina Gabri Nicoletti, Salvetti Marco, Baione Viola, Patti Francesco, Alessandra Girolama Marfia, Sibilia Grazia, Scarano Valentina, Orlando Davide, Stabile Giovanni, Tedeschi Gioacchino, Antonio Gallo

**Affiliations:** 1 MS Center-I Division of Neurology, Department of Advanced Medical and Surgical Sciences, Università degli Studi della Campania “Luigi Vanvitelli”, Napoli, Italia, piazza Miraglia, 2, 80138 Naples, Italy; 2 Department of Neurosciences, Mental Health and Sensory Organs, Sapienza University, Roma, Italy; 3 Fondazione Policlinico Universitario A. Gemelli IRCCS and, Università Cattolica del Sacro Cuore, Roma, Italy; 4 Department of Human Neurosciences, Sapienza University of Rome, Roma, Italy; 5 Department GF Ingrassia, University of Catania, Catania, Italy; 6 Multiple Sclerosis Clinical and Research Unit, Department of Systems Medicine, Tor Vergata University and Hospital, Roma, Italy; 7 UOC di Neurologia e Centro Sclerosi Multipla, Ospedale San Paolo, ASL Napoli 1 Centro, Napoli, Italy; 8 UOC di Neurologia e Centro Sclerosi Multipla, Azienda Ospedaliera San Giuseppe Moscati, Avellino, Italy; 9 Centro Sclerosi Multipla, Presidio Ospedaliero “San Giuseppe Moscati”, ASL Caserta, Aversa, CE Italy; 10 UOD Endoscopia Digestiva, Azienda Ospedaliero-Universitaria Sant’Andrea, Roma, Italy

**Keywords:** Multiple sclerosis, Bowel, Symptoms, Multicenter study, Gut dysfunction

## Abstract

**Introduction:**

Bowel dysfunction (BD) is reported as a common and disabling symptom in multiple sclerosis (MS) patients. To date, no studies have explored the prevalence of these symptoms in a large multicenter outpatient setting. The aims of the present study are to assess: (i) the prevalence of BD in a large multicenter Italian MS population, and (ii) the correlation between clinico-demographic variables and the severity of BD.

**Methods:**

Each of the nine participating center screened MS patients prospectively: 1100 subjects were enrolled. All patients underwent the Expanded Disability Status Scale (EDSS) and completed the Neurogenic Bowel Dysfunction score (NBDs). Multivariable linear and logistic regression models were used to assess the association between NBDs and several clinico-demographic variables.

**Results:**

Fourteen percent of MS patients showed a moderate/severe BD (NBDs > 10); this percentage increased in patients with high disability, ranging from 26 to 32%. Moderate/severe BD was more frequent in MS patients with: progressive phenotypes, higher disability, older age, and longer disease duration. NBDs severity was predicted by female sex, ambulation impairment and bladder symptoms.

**Conclusion:**

This study confirms the relatively high prevalence of moderate/severe BD in a large, multicenter, unselected, outpatient MS population. BD appears to be mainly associated to female sex and MS-related disability.

## Introduction

Neurogenic bowel dysfunction (NBD) is a common and disabling feature of multiple sclerosis (MS) and appears to be associated with the presence of bladder dysfunction, high level of disability, and long disease duration[1], even if it is described also in early phase of the disease [2, 3].

The presence of NBD impacts on quality of life (QoL), daily activities and employment status of MS patients [1].

Many factors contribute to the onset of NBD in MS. Polypharmacy, physical disability and comorbidities are relevant indirect factors which combines with the direct effect of MS on both autonomic and voluntary control of the bowel, mainly secondary to spinal cord involvement [4]. NBD in MS includes both constipation and/or fecal incontinence symptoms, arising from a complex pathophysiology including slow gut transit, pelvic floor dyssynergia and/or ano-rectal hyposensitivity [5]. Although the detrimental effects of bowel symptoms, they are rarely investigated and frequently unrecognized and undertreated. [6]

The prevalence of NBD in MS is estimated between 39 and 73%, depending on the studied population, making the real quantification of the phenomenon difficult.

[1] In particular, constipation is reported in 17–94%, while fecal incontinence in 1–69% of MS population [7]. Quite frequently, constipation and incontinence coexist and alternate in the same patient [8].

The wide range of prevalence reported in previous studies might reflect differences in sample size, recruitment criteria, disease duration, level of disability, definition of NBD and other methodological aspects (i.e. patient self-assessment *vs* validated scoring system) [7].

To our knowledge, no study has explored the prevalence of NBD symptoms in a large multicenter setting by means of Neurogenic bowel dysfunction score (NBDs), a validated scoring system for neurogenic bowel dysfunction [9], previously applied in MS [4, 5].

Against this background, the objective of the present study was to investigate (i) the prevalence of bowel symptoms in a large, unselected multicenter Italian MS population, using validated questionnaire for NBD [9], that allows to rapidly (few minutes) explore both continence and constipation symptoms; ii) the association between the severity of these symptoms and clinico-demographic variables.

## Methods

### Study population

One thousand and one hundred sixty MS patients (Table 1) [10] were consecutively enrolled in one month at outpatient clinics of nine Italian MS centers, which included: (a) I Clinica Neurologica, Dipartimento di Scienze Mediche e Chirurgiche Avanzate, Università degli Studi della Campania “Luigi Vanvitelli”, Napoli; (b) Dipartimento di Neuroscienze, Salute mentale ed organi di senso—Università La Sapienza—Ospedale Sant’Andrea, Roma; (c) Fondazione Policlinico A Gemelli, IRCSS, Università Cattolica del Sacro Cuore, Roma; (d) Centro Sclerosi Multipla, Policlinico Umberto I, Roma; (e) Dipartimento G. F. Ingrassia, Università di Catania, Catania; (f). Centro Sclerosi Multipla, Dipartimento di Medicina dei Sistemi, Università degli Studi di Roma Tor Vergata, Roma, Italia; (g) UOC di Neurologia e Centro Sclerosi Multipla, Ospedale San Paolo—ASL Napoli 1 Centro, Napoli; (h) UOC di Neurologia, Azienda Ospedaliera San Giuseppe Moscati, Avellino, Italia; (i) Centro Sclerosi Multipla; Ospedale San Giuseppe Moscati; Aversa (CE).

**Table 1:** Main demographic and clinical characteristics of patients with multiple sclerosis (MS) enrolled in the study

MS patients (*n* = 1100)	
Mean age, years (SD)	44.2 (12.3)
Sex, number (%) M/W	376 (34)/724 (66)
Mean disease duration, years (SD)	11.2 (8.7)
Median EDSS (range)	2 (0.0–9.0)
Phenotype—RR/SP/PP (%)	927/118/55 (84%/11%/5%)
NBDs—mean, median (SD, range)	3.8, 1 (5.5, 0–33)
IPSS—mean, median (SD, range)	10.7, 7 (10.1, 0–35)

*MS* multiple sclerosis, *SD* standard deviation, *M* men, *W* women, *EDSS* Expanded Disability Status Scale, *RR* relapsing remitting, *SP* secondary progressive, *PP* primary progressive, *NBDs* Neurogenic bowel dysfunction score, *IPSS* International Prostatic Symptoms Score

Inclusion criteria were: (1) diagnosis of MS [10]; (2) age ≥ 18 years; (3) ability to provide informed consent and to provide either verbal or written responses to the study questionnaires.

From the initial sample enrolled (1162 patients), 62 patients were excluded for incomplete/wrong compilation of questionnaires. The final sample involved 1100 patients. Fourteen patients refused to be enrolled in the study.

The study was conducted performed in accordance with the ethical standards laid down in the 1964 Declaration of Helsinki and its later amendments and approved by the local Ethic Committee of each Center (Ethic Committee of coordinator center: Comitato etico—Azienda Ospedaliera Universitaria Università degli studi della Campania “Luigi Vanvitelli”) and a signed informed consent was obtained from all participants.

### Clinical characteristics

All enrolled subjects underwent a neurological examination including the Expanded Disability Status Scale (EDSS) [11] and completed the Italian version of the following questionnaires: NBDs [9] and the International Prostatic Symptoms Score (IPSS) [12].

NBDs consists of ten simple questions—each with a different weighted score—that explore and quantify NBD symptoms, including both constipation and incontinence ones. The combination of response (total score) creates four levels of severity of dysfunction: 0–6 very minor, 7–9 minor, 10–13 moderate, and 14–47 severe.

IPSS is a seven‐question screening tool (quantifying incomplete emptying, frequency, intermittency, urgency, weak stream, straining and nocturia), originally designed to assess storage and voiding symptoms in benign prostatic hyperplasia, but which has been largely used also to score neurogenic bladder-associated symptoms. Each question scores from 1 to 5 for a maximum total of 35 points with three different levels of severity dysfunction: 0–7: minor; 8–19: moderate; 20–35: severe.

### Statistical analysis

Descriptive statistics were calculated to describe the characteristics of the population. For categorical variables, absolute frequencies and percentages were reported, while mean, median, standard deviation, inter-quantile range were calculated for continuous variables. Shapiro–Wilk test was performed to examine normality of the data. To evaluate the association between NBDs (dichotomous outcome categorized in: very minor-minor NBDs, moderate-severe NBDs) and other characteristics, the Fisher exact test and Wilcoxon rank-sum test (aka Mann–Whitney test) were used accordingly. Bonferroni method was used to adjust pairwise comparisons after a comparison of proportions using a Fisher’s exact test. A backward selection with a significance level threshold of 0.1 was used to identify an appropriate subset of independent variables to be included in the model. Multivariable linear regression was conducted to study the association between NBDs score and sex, antispastic drugs, neurogenic bladder drugs, IPSS, Sphinteric FS EDSS, Ambulation EDSS. Multivariable logistic regression was conducted to analyze the association between NBDs as dichotomous outcome and IPSS, Sphinteric FS EDSS, Ambulation EDSS. Beta coefficients and 95% CI, and OR and 95% CI were reported, respectively, for multivariable linear and logistic regression. In all the analyses, a *p* value < 0.05 was considered as statistically significant.

### Subgroup analysis

Stratification of patients based on NBDs score was made in patients with EDSS ≥ 4, ≥ 5 and ≥ 6. Percentage of patients on symptomatic treatments was calculated in patients with EDSS ≥ 5 and EDSS ≥ 10.

## Results

### Clinical-demographic data

Table 1 summarizes the main clinical-demographic data.

The patients enrolled assumes the following treatment at moment of the administration of questionnaires:

- Disease modifying drugs (DMDs) (number of patients on this treatment, percentage on total of patients): Alemtuzumab (28, 2.6%); Cladribine (5, 0.5%); Dimethyl Fumarate (184, 16.7%); Fingolimod (122, 11.1%); Glatiramer Acetate (73, 6.4%); IFNBeta (150, 13.5%); Natalizumab (135, 12.3%); Ocrelizumab (46, 4.2%); Teriflunomide (76, 6.9%); Others (24, 2.45%); No DMDs (254, 23.1%).
- Symptomatic treatments (number of patients on this treatment, percentage on total of patients): antispastics (116, 11.2%); antidepressants (130, 12.6%); anxiolytics (71, 6.9%); neurogenic bladder Drugs (145, 14.1%); neuropathic pain drugs (118, 11.5%); physiotherapy (244, 23.6%).

### Stratification of patients by NBDs (Fig. 1)

**Fig. 1:**
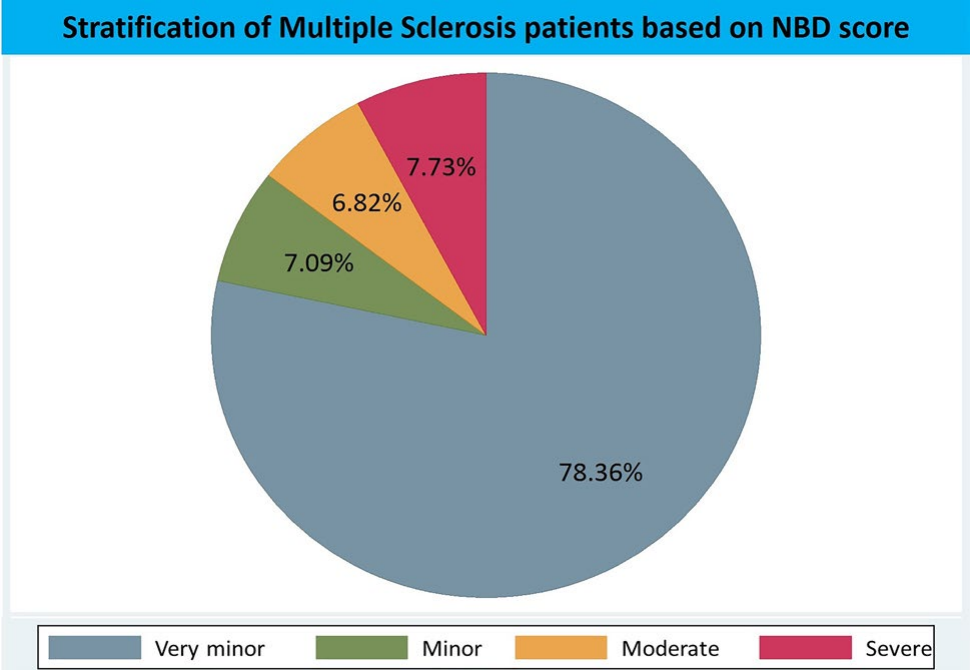
Stratification of Multiple Sclerosis patients based on NBDs. *NBDs* Neurogenic Bowel Dysfunction score

Very minor (0–6) 78.4% (number of patients = 862), minor (7–9) 7.1% (number of patients = 78), moderate (10–13) 6.8% (number of patients = 75), severe (≥ 14) 7.7% (number of patients = 85).

MS patients with a NBDs moderate–severe (≥ 10), compared to those with a NBDs very minor–minor were older, with longer disease duration, more frequently with a progressive MS phenotype and with a higher EDSS and IPSS (Table 2).

**Table 2:** Comparison between multiple sclerosis patients subgroups: very minor-minor NBDs *vs* moderate-severe NBDs

MS patients (*n* = 1100)	NBDs < 10 (*n* = 940, 83%)	NBDs ≥ 10 (*n* = 160, 17%)	*p*
*Age*, mean years (SD)	43.7 (12.5)	47.2 (11.0)	0.0006^b^
*Sex*, M/W Number (%)	325 (35%) /615 (65%)	109 (68%)/51 (32%)	n.s^a^
*Disease duration*, mean years (SD)	10.9 (8.9)	12.7 (7.7)	0.0002^b^
*EDSS*, median (range)	2 0–9	4 (0–9)	< 0.0001^b^
*Phenotype RR/SP/PP* Number (%)	819 (87%)/83 (9%)/38 (4%)	108 (68%)/35 (22%)/17 (10%)	< 0.0001^a^
*IPSS* mean, median (SD, range)	8.9, 6 (8.9; 0–35)	21.5, 23 (9.9; 0–35)	< 0.0001^b^

Bonferroni Method Correction

*MS* multiple sclerosis, *NBDs* Neurogenic bowel dysfunction score, *SD* standard deviation, *M* men, *W* women, *EDSS* Expanded Disability Status Scale, *RR* relapsing remitting, *SP* secondary progressive, *PP* primary progressive, *IPSS* International Prostatic Symptoms Score

^a^Fisher test

^b^Wilcoxon rank-sum test (Mann–Whitney)

### Subgroup analysis

**Fig. 2:**
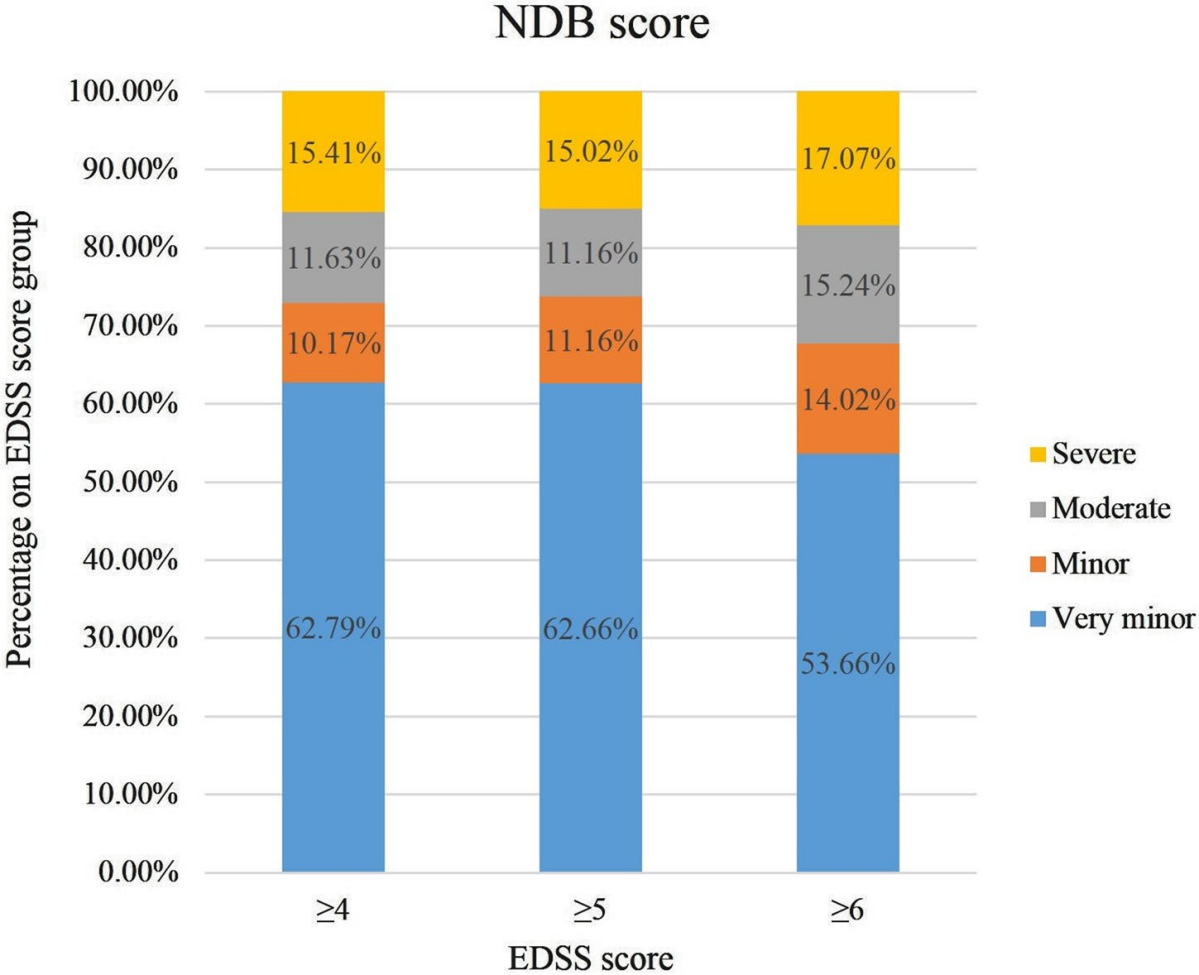
Stratification of Multiple Sclerosis patients with EDSS score ≥ 4, ≥ 5 and ≥ 6 (on class population) based on NBDs. *EDSS* Expanded Disability Status Scale, *NBDs* Neurogenic Bowel Dysfunction score

- Stratification patients with EDSS score ≥ 4, ≥ 5 and ≥ 6 (on class population) based on NBDs (Fig. 2):
  - EDSS ≥ 4 (344 patients): very minor (0–6) 62.8% (number of patients = 216), minor (7–9) 10.2% (number of patients = 35), moderate (10–13) 11.6% (number of patients = 40), severe (≥ 14) 15.4% (number of patients = 53).
  - EDSS ≥ 5 (233 patients): very minor (0–6) 62.7% (number of patients = 146), minor (7–9) 11.2% (number of patients = 26), moderate (10–13) 11.2% (number of patients = 26), severe (≥ 14) 15% (number of patients = 35).
  - EDSS ≥ 6 (164 patients): very minor (0–6) 53.7% (number of patients = 88), minor (7–9) 14% (number of patients = 23), moderate (10–13) 15.2% (number of patients = 25), severe (≥ 14) 17.1% (number of patients = 28).
- Symptomatic treatments (number of patients on this treatment, percentage on total of patients) in patients with EDSS ≥ 5 and NBD ≥ 10:
  - EDSS ≥ 5 (233 patients): antispastics (78, 33.5%); antidepressants (57, 24.5%); anxiolytics (24, 10.3%); neurogenic bladder Drugs (58, 24.18%); neuropathic pain drugs (50, 21.5%); physiotherapy (134, 57.5%).
  - NBD ≥ 10 (160 patients): antispastics (36, 22.5%); antidepressants (28, 17.5%); anxiolytics (17, 10.6%); neurogenic bladder Drugs (71, 44.37%); neuropathic pain drugs (37, 23.1%); physiotherapy (76, 47.5%).

### Multivariable linear regression between NBDs and clinico-demographic variables (Table 3)

**Table 3:** Multivariable linear regression coefficients (*B*) with 95% CI for neurogenic bowel dysfunction score (NBSs) adjusted by sex, antispastic drugs, neurogenic bladder drugs, IPSS, Sphinteric FS EDSS, Ambulation EDSS

Parameter	*B*	SE	95% Wald confidence interval		Hypothesis test		
			Lower	Upper	Wald Chi-square	*df*	Sig
(Intercept)	1.89	0.582	0.75	3.03	10.55	1	0.001
SEX = M (F ref)	− 0.61	0.31	− 1.22	− 0.004	3.89	1	0.048
Antispastic drugs	− 1.44	0.54	− 2.49	− 0.38	7.17	1	0.007
Neurogenic bladder drugs	1.98	0.51	0.99	2.98	15.21	1	< 0.001
IPSS	0.12	0.02	0.074	0.16	30.29	1	< 0.001
Sphinteric FS EDSS	1.61	0.22	1.19	2.03	55.78	1	< 0.001
Ambulation EDSS	0.30	0.082	0.14	0.46	13.30	1	< 0.001
Likelihood ratio Chi-square: < 0.0001							

*NBDs* Neurogenic bowel dysfunction score, *SE* standard error, *M* men, *F* female, *IPSS* International Prostatic Symptoms Score, *FS* functional system, *EDSS* Expanded Disability Status Scale

NBDs was associated (*R*^2^ = 0.3317) to (i) sphinteric functional system (FS) (*p* < 0.001) and ambulation scores (*p* < 0.001) of EDSS, (ii) IPSS (*p* < 0.001) and (iii) to use of drugs for neurogenic bladder (*p* < 0.001). Male sex (*p* = 0.048) and use of antispastic drugs (*p* = 0.007) seemed to be protective.

### Multivariable logistic regression between binary NBDs (< or ≥ 10) and clinico-demographic variables (Table 4)

**Table 4:** Multivariable logistic regression odds ratio (OR) with 95% CI for neurogenic bowel dysfunction score (NBDs) adjusted by IPSS, Sphinteric FS EDSS, Ambulation EDSS

Outcome variable: Binary NBDs (< or ≥ 10)						
Parameter	OR	SE	*p* value	[95% confidence interval]		*p* value
IPSS	1.07	0.01	< 0.001	1.05	1.09	*p* < 0.001
Sphinteric FS EDSS	2.02	0.24	< 0.001	1.60	2.56	*p* < 0.001
Ambulation EDSS	1.12	0.05	< 0.001	1.08	129	*p* < 0.001

*NBDs* Neurogenic bowel dysfunction score, *OR* ODDS Ratio, *SE* standard error, *IPSS* International Prostatic Symptoms Score, *FS* functional system, *EDSS* Expanded Disability Status Scale

The risk to have a NBDs ≥ 10 was associated (Pseudo *R*^2^ = 0.342) to IPSS (OR 1.07; *p* < 0.001), sphinteric FS (OR 2.02, *p* < 0.001) and ambulation score (OR 1.12, *p* < 0.001) of EDSS.

## Discussion

The main findings of the present study was that 14.5% of the studied MS population suffered of moderate-severe NBD symptoms.

This is partially in contrast with previous frequencies reported in reviews and meta-analyses ranging from 39 to 73% [1] and 6–52%[7]. The observed differences in the prevalence of NBD symptoms might be explained in several ways, including differences in (i) sample size (most previous studies had small sample sizes: < 100 subjects) [7], (ii) patients’ inclusion/exclusion criteria (e.g. enrolling only patients that referred bowel discomforts [4]), (iii) data collection (validated scales vs patient self-assessment vs physiologic markers), and (iv) studied populations (i.e. MS phenotypes, disease duration, disability, etc.) [7].

It is evident that our population was predominantly composed by RRMS patients (84%) with a low disability. This might help to explain our results. However, it is worth remembering that bowel symptoms can be present regardless of disease duration, type, or level of disability [1].

Another thing to emphasize is that our data derived by a large number of unselected MS patients, enrolled in a multicenter outpatients setting. If on the one hand this might have limited the enrollment of more disabled patients, on the other hand the studied population should more closely represent the typical real-life outpatient MS population, encountered everyday by MS specialists at tertiary centers.

As abovementioned, because our sample was characterized by a low disability (median EDSS = 2), we run a subgroup analysis on patients with high EDSS: ≥ 4, ≥ 5, and ≥ 6. Predictably, in these subgroups the percentage of MS patients with moderate-severe NBD symptoms ranged from 26 to 32%, in line with previous studies. [1, 7] Similarly, the percentage of patients assuming symptomatic treatments in patients with higher EDSS (≥ 5) and those with moderate-severe NBD symptoms was higher.

When we stratified our sample based on the level of NBD symptoms, in line with previous studies [1, 4, 6, 13, 14], we found that MS patients reporting higher NBDs (≥ 10 = moderate to severe symptoms) were older, with longer disease duration, higher disability, and more frequently with progressive disease. Moreover, as expected [1, 6, 13, 14], bowel symptoms were frequently associated with those of neurogenic bladder.

To weight the contribution of clinico-demographic characteristics to explain NBD symptoms in MS patients, we performed two regression models (one with total NBD scores and one with a binarized NBD score as dependent variables). Both the models showed that the main factors predicting the presence of bowel symptoms in MS patients were the level of physical disability, as measured by EDSS, and the severity of neurogenic bladder symptoms.

The level of disability has already been demonstrated to be one of the most relevant independent factors related to the presence of bowel symptoms [1, 14, 15]. Since EDSS-measured disability is strongly related to ambulation deficits, the observed association between EDSS and NBDs might be a consequence of immobilization, which brings a greater tendency to constipation. A higher physical disability can limit the ability to access the toilet, determining behavioral modifications such as learning to be constipated to avoid too move to the bathroom, or, conversely, can become an impediment to reach a toilet in time, with episodes of fecal incontinence [16]. Moreover, both ambulation impairment [17] and NBD are associated with a more relevant spinal cord involvement [4, 16]. The spinal cord plays a central role in bowel function, as previously demonstrated in patients with spinal cord injury and with MS [4, 16]. In MS, the reduction/absence of central modulation of spinal reflex activity may cause autonomic dysfunction of gut peristalsis, determining constipation or a reduced inhibition of parasympathetic output, with uncontrolled colonic contractions and incontinence [4].

Results regarding the association between NBDs and neurogenic bladder symptoms were also in line with previous studies [13, 14, 18–20] and confirm the existence of a common pathophysiology related to the spinal cord MS-related damage [4, 16, 19, 21]

Factors associated to lower NBDs were male sex and, quite surprisingly, the use of antispastic drugs. While it has been showed that being a woman increases the risk of experiencing NBD symptoms and this has been related—amongst other factors—to parity [4, 14, 22], the use of antispastic drugs is expected to favorite constipation due to their anticholinergic effect [19]. A possible interpretation of our result, however, is that, despite the pharmacodynamics of such drugs, their positive effect on ambulation impairment might ameliorate bowel motility and function, also reducing the abovementioned harmful behaviors put in place by MS patients with ambulatory limitations.

This work is not without limitations. First, it is cross-sectional. Second, our population has been enrolled in an outpatient setting: this might explain the lower prevalence of patients with high disability and NBD respect to previous studies. Third, we have not studied constipation and incontinence separately: this point should be addressed in future studies.

In conclusion, the present multicenter study suggests that NBD symptoms might be less frequent in MS patients respect to previous reports. Factors that might explain our results, other than the low average disability of our population, might be: (i) the beneficial effect of the Mediterranean diet—rich in fibers and omega 3—on bowel microbiota and function [23] 24, and (ii) the high percentage of patients on physiotherapy (23.4% of the entire population and 57% of patients with high disability). Coupling the Mediterranean diet with exercise has been suggested as a possible primary intervention in individuals with NBD to reduce gut dysbiosis as well as NBD symptoms. [24]

It is worthy to note that a prevalence of about 14% of moderate–severe NBD is quite significant in a population composed mainly by young, active, low-disabled MS patients. On the other hand, when we analyze a subgroup of patients with high levels of disability the prevalence of moderate–severe NBD is higher (26–32%), confirming previous data in similar populations.

MS specialists should systematically and proactively screen/check for NBD symptoms in clinical practice, since these latter are too often considered a taboo topic by both patients and physicians, [1] such that the large majority of MS patients tend to do not refer these symptoms and passively accept them. The proactive screening should be extended also to young MS patients with low disability, taking into account that moderate to severe constipation has been reported also as an early symptom, or even a prodrome, of MS [2, 3].

A brief questionnaire, such as the NBDs, offers the opportunity to rapidly and reliably explore, quantify and monitoring both constipation and incontinence symptoms and their impact on quality of life in an outpatient setting. A routine screening of bowel dysfunction in MS might allow early identification and management of patients suffering of these disabling symptoms. This is relevant to put in place a prompt and tailored management of these symptoms to prevent their worsening and reduce their impact of patients QoL.

## Data Availability

Not applicable.
