# Correction to: Prevalence and predictors of bowel dysfunction in a large multiple sclerosis outpatient population: an Italian multicenter study

**DOI:** 10.1007/s00415-022-11083-1

**Published:** 2022-03-27

**Authors:** Alvino Bisecco, Arianna Fornasiero, Assunta Bianco, Antonio Cortese, Emanuele D’Amico, Giorgia Mataluni, Leonardo Sinisi, Daniele Spitaleri, Renato Docimo, Maria Chiara Buscarinu, Massimiliano Mirabella, Sebastiano Giuseppe Crisafulli, Aurora Zanghì, Carolina Gabri Nicoletti, Marco Salvetti, Viola Baione, Francesco Patti, Alessandra Girolama Marfia, Grazia Sibilia, Valentina Scarano, Davide Orlando, Giovanni Stabile, Gioacchino Tedeschi, Antonio Gallo

**Affiliations:** 1 MS Center‑I Division of Neurology, Department of Advanced Medical and Surgical Sciences, Universita degli Studi della Campania “Luigi Vanvitelli”, Napoli, Italia, piazza Miraglia, 2, 80138 Naples, Italy; 2 Department of Neurosciences, Mental Health and Sensory Organs, Sapienza University, Roma, Italy; 3 Fondazione Policlinico Universitario A. Gemelli IRCCS and, Universita Cattolica del Sacro Cuore, Roma, Italy; 4 Department of Human Neurosciences, Sapienza University of Rome, Roma, Italy; 5 Department GF Ingrassia, University of Catania, Catania, Italy; 6 Multiple Sclerosis Clinical and Research Unit, Department of Systems Medicine, Tor Vergata University and Hospital, Roma, Italy; 7 UOC di Neurologia e Centro Sclerosi MultiplaOspedale San Paolo, ASL Napoli 1 Centro, Napoli, Italy; 8 UOC di Neurologia e Centro Sclerosi MultiplaAzienda Ospedaliera San Giuseppe Moscati, Avellino, Italy; 9 Centro Sclerosi Multipla, Presidio Ospedaliero “San Giuseppe Moscati”, ASL Caserta, Aversa, CE Italy; 10 UOD Endoscopia DigestivaAzienda Ospedaliero-Universitaria Sant’Andrea, Roma, Italy

## Correction to: Journal of Neurology (2022) 269:1610–1617 10.1007/s00415-021-10737-w

The original version of this article unfortunately contained a mistake. The given and family names of authors were Interchanged except the following author Maria Chiara Buscarinu, Carolina Gabri Nicoletti, Alessandra Girolama Marfia and Antonio Gallo

The correct author names should be

**Table:** 

Given Name	Family Name
Alvino	Bisecco
Arianna	Fornasiero
Assunta	Bianco
Antonio	Cortese
Emanuele	D’Amico
Giorgia	Mataluni
Leonardo	Sinisi
Daniele	Spitaleri
Renato	Docimo
Massimiliano	Mirabella
Sebastiano Giuseppe	Crisafulli
Aurora	Zanghì
Marco	Salvetti
Viola	Baione
Francesco	Patti
Grazia	Sibilia
Valentina	Scarano
Davide	Orlando
Giovanni	Stabile
Gioacchino	Tedeschi

